# *IntelliGenes*: a novel machine learning pipeline for biomarker discovery and predictive analysis using multi-genomic profiles

**DOI:** 10.1093/bioinformatics/btad755

**Published:** 2023-12-14

**Authors:** William DeGroat, Dinesh Mendhe, Atharva Bhusari, Habiba Abdelhalim, Saman Zeeshan, Zeeshan Ahmed

**Affiliations:** Rutgers Institute for Health, Health Care Policy and Aging Research, Rutgers, The State University of New Jersey, New Brunswick, NJ 08901, United States; Rutgers Institute for Health, Health Care Policy and Aging Research, Rutgers, The State University of New Jersey, New Brunswick, NJ 08901, United States; Rutgers Institute for Health, Health Care Policy and Aging Research, Rutgers, The State University of New Jersey, New Brunswick, NJ 08901, United States; Rutgers Institute for Health, Health Care Policy and Aging Research, Rutgers, The State University of New Jersey, New Brunswick, NJ 08901, United States; Rutgers Cancer Institute of New Jersey, Rutgers University, New Brunswick, NJ 08901, United States; Rutgers Institute for Health, Health Care Policy and Aging Research, Rutgers, The State University of New Jersey, New Brunswick, NJ 08901, United States; Department of Medicine, Robert Wood Johnson Medical School, Rutgers Health, New Brunswick, NJ 08901, United States

## Abstract

**Summary:**

In this article, we present *IntelliGenes*, a novel machine learning (ML) pipeline for the multi-genomics exploration to discover biomarkers significant in disease prediction with high accuracy. *IntelliGenes* is based on a novel approach, which consists of nexus of conventional statistical techniques and cutting-edge ML algorithms using multi-genomic, clinical, and demographic data. *IntelliGenes* introduces a new metric, i.e. Intelligent Gene (I-Gene) score to measure the importance of individual biomarkers for prediction of complex traits. I-Gene scores can be utilized to generate I-Gene profiles of individuals to comprehend the intricacies of ML used in disease prediction. *IntelliGenes* is user-friendly, portable, and a cross-platform application, compatible with Microsoft Windows, macOS, and UNIX operating systems. *IntelliGenes* not only holds the potential for personalized early detection of common and rare diseases in individuals, but also opens avenues for broader research using novel ML methodologies, ultimately leading to personalized interventions and novel treatment targets.

**Availability and implementation:**

The source code of *IntelliGenes* is available on GitHub (https://github.com/drzeeshanahmed/intelligenes) and Code Ocean (https://codeocean.com/capsule/8638596/tree/v1).

## 1 Introduction

Multi-genomic data, including whole genome sequencing (WGS) and RNA-seq of transcribed genes, informs us of a patient’s inherent genetic makeup with the most comprehensive view of the genome (Zeeshan *et al.* 2020). WGS-based gene variant detection when combined with RNA-seq-driven gene expression, and clinical and demographic information has the potential to reveal novel and sensitive biomarkers and stratify patient populations based on their disease risk (Vadapalli *et al.* 2022). To improve the deciphering of common and rare disease factors, we need to deeply investigate known and identify novel genes that are responsible for the development of disease. Gene Expression Analysis (GEA) and Genome-wide association studies (GWAS) have remarkably assisted in understanding the genetic basis of human disease by uncovering millions of loci associated with various complex phenotypes (Visscher *et al.* 2017). However, these are unable to identify multi-genomic profile-based biomarkers and predict disease at high accuracy. These limitations are not exclusive to GWAS and GEA, as no method or technology to date can identify all the genetic components of complex traits (Altshuler *et al.* 2008). In addition, a persistent challenge in multi-genomic data analysis lies in the handling, integration, and standardization of large volumes of sequencing data. Several multi-genomics approaches demonstrate the potential of investigating genes associated with disease. However, the current and still unresolved challenges include the unavailability of bioinformatics and biostatistics applications to greatly enhance the performance of analysis as well as understand the dimensions and complexity of multi-genomics data (Ahmed 2022).

Studying genetic insight with the application of Artificial Intelligence (AI), Machine Learning (ML), and state-of-the-art bioinformatics approaches will improve the processes of discovering disease causing variants and decode genetics of complex phenotypes to predict, prevent, and treat complex diseases. These can play a vital role in the recognition, extraction, and prediction of nonlinear structures and biological patterns, which can decode the genetics of complex phenotypes (Ahmed *et al.* 2020). This will support discovering genotype-phenotype associations by linking multi-genomic, clinical, and demographic data. ML offers multiple supervised and unsupervised algorithms, which can be used to analyze multi-genomic, clinical, and demographic data with the potential for learning from a continuum of dataset displaying heterogeneous levels of granularity. However, the important question here is, which ML algorithm is appropriate for a proposed clinical/scientific problem? Choosing the right ML model can have a huge impact on the accuracy of the predicted outcome and biomarker discovery. Classifying tasks based on available predictor variables can be a key step to correctly addressing the problem of choosing a suitable ML algorithm (Ahmed *et al.* 2020, Vadapalli *et al.* 2022).

Recently, we have published an important study in the Briefing in Bioinformatics (Vadapalli *et al.* 2022), reporting evaluation and comparative analysis of various ML approaches using the gene-variant and expression data for statistical and predictive analysis of a wide variety of disorders. Our study concluded that the Support Vector Machine (SVM) and Random Forest (RF) are the most applied and successful ML algorithms used to make high accuracy predictions and solve regression and classification problems. The major differences between these two include adjusting hyperparameters (a parameter whose value is used to control the learning process) in SVM to prevent over and underfitting compared to no adjustment in RF. SVM has been implemented to distinguish genetic susceptibility factors and identify previously unknown features that corresponded to common disease (Isakov *et al.* 2017, Maniruzzaman *et al.* 2019), when RF has been applied to identify differentially expressed genes played an important role in disease prognosis by acting as a potential biomarker (Kegerreis *et al.* 2019, Schaack *et al.* 2021, Zhao *et al.* 2021). Other than these two algorithms, we found that some other widely used approaches and that include Xtreme Gradient Boosting Decision Trees (XGBoost), Logistic regression (LR), Naïve Bayes, Decision tree, Artificial neural network, k-nearest neighbor (k-NN), and Analysis of Variance (ANOVA), Adaboost, Gradient Boosting, Linear discriminant analysis, Quadratic discriminant analysis, Gaussian process classification, and Clustering (Vadapalli *et al.* 2022). These approaches are suitable for solving different kinds of data analytic problems and predictive analysis using genomic data of variable types and sizes (Vadapalli *et al.* 2022). However, there is no single peer reviewed approach, which can fit for all or at least suitable for multiple situations. An ML pipeline, assembling appropriate statistical and ML approaches can be a solution to overcome the limitations of singular approaches.

## 2 Materials and methods

**Figure 1.**
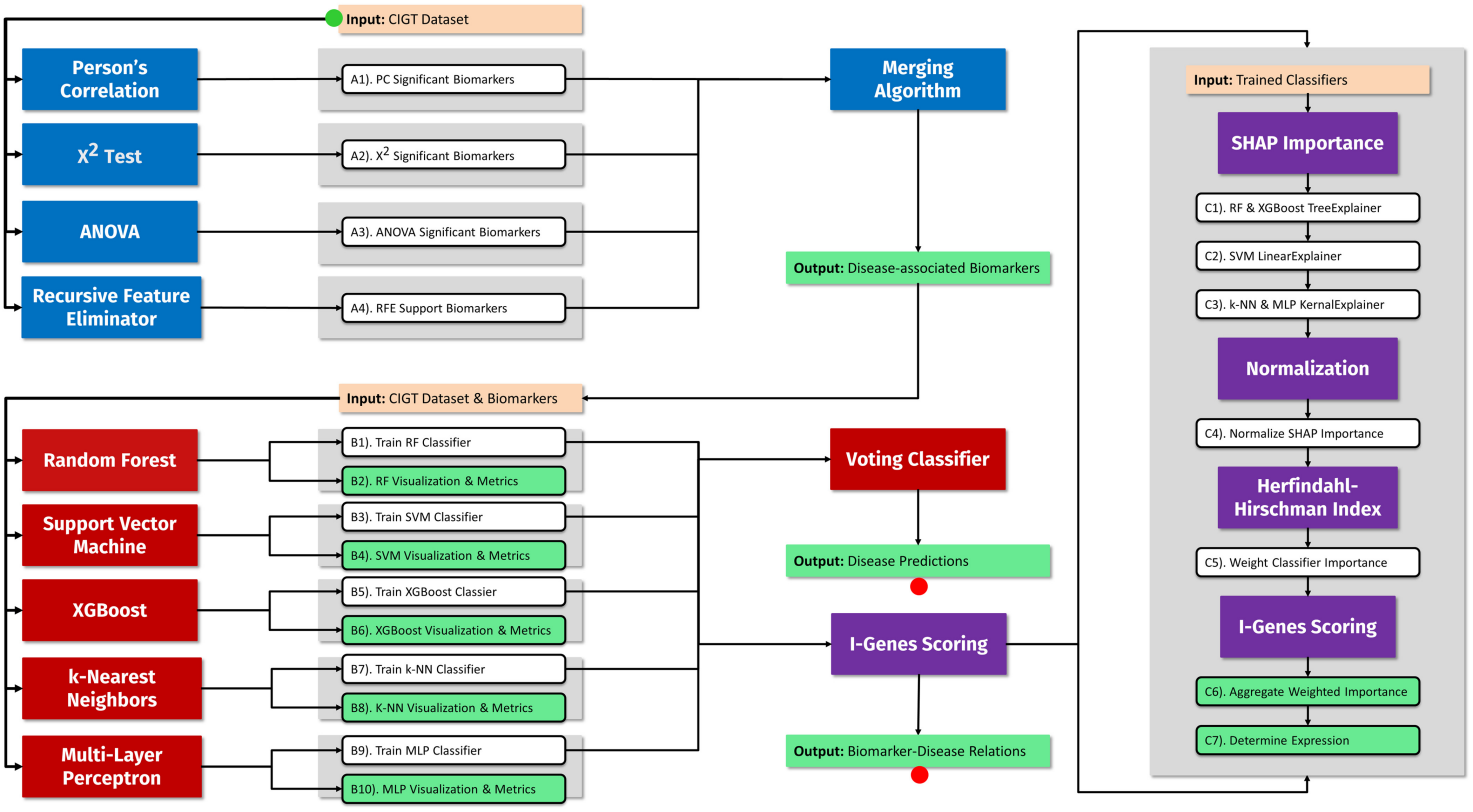
*IntelliGenes* methodology.

In this article, we propose a novel ML approach that involves harnessing transcriptomic data, along with demographic and clinical information (Fig. 1). We have designed and developed a new ML pipeline, i.e. *IntelliGenes*, that employs a unique combination of classical statistical methods and state-of-the-art ML algorithms to identify novel biomarkers and predict disease in individuals. Using a nexus of ML algorithms, our approach can uncover information that usually goes undetected by classical statistics and traditional bioinformatics techniques. Input to the overall methodology is the AI/ML ready data in the Clinically Integrated Genomics and Transcriptomics (CIGT) format, including information about patient’s age, gender, racial and ethnic background, diagnoses, and RNA-seq driven gene expression data. These attributes have proven to be ideal for the development of studies integrating genotype and phenotype (Wilczewski *et al.* 2023).

We implemented three classical statistics (Pearson correlation, Chi-square test, and ANOVA) and one ML classifier (Recursive Feature Elimination) to extract significant disease-associated biomarkers from a patient cohort. Then, seven ML classifiers (RF, SVM, XGBoost, k-NN, Multi-Layer Perceptron, a soft voting classifier, and a hard voting classifier) are applied to compute top percentage (e.g. 10%) profiles of multi-genomic data-based profiles and rank them to predict a diagnosis, in a unique patient, with the highest accuracy. Just as with our selection algorithms, users have the flexibility to combine various classifiers to tailor their ideal methodology. Disease prediction with *IntelliGenes* requires a list of biomarkers and a training dataset to make patient predictions. The resulting output includes individual patient predictions, various classifier metrics, and customizable user visualizations.

*IntelliGenes* introduces the novel metric of I-Gene Score that measures the importance of individual biomarkers to disease prediction. Calculating I-Genes Score is the fundamental component of *IntelliGenes*, fully categorizing a transcriptomic feature’s role in a disease. Using Shapley Additive exPlanations (SHAP) and Herfindahl-Hirschman Indexes (HHI), I-Genes Scores determine the weighted usefulness of biomarkers and characterize their expression in biological systems. The SHAP values assign importance to the features used in disease prediction, and HHI weighs classifiers’ reliance on individual high-impact biomarkers. Classifiers, where fewer biomarkers are responsible for high-accuracy predictions, receive a greater weight downstream. SHAP scores normalize and aggregate according to these weights, resulting in our I-Gene score. For example, the SHAP importance of biomarkers in classifiers where a sole biomarker is responsible for predictions will be given more weight in the final I-Gene score calculation. HHI measures concentration, differing from methods like the Gini coefficient, which measures inequality. Examining concentration more accurately captures the patterns in which classifiers make disease predictions. HHI weights are multiplied to normalized importance, which is then summed across all classifiers in the ensemble model for each feature. I-Gene score-based profiles contain the direction of a gene’s expression in a disease. Using SHAP once, we can examine what types of features contribute to positive disease classifications. If attributes contributing positively to these classifications are typically lower in control patients than case patients, we deem that feature overexpressed.

The I-Gene score includes directionality, helping researchers utilizing *IntelliGenes* understand if biomarker overexpression or under expression contributes to disease. *IntelliGenes* is user-friendly, portable, and a cross-platform application, compatible to Microsoft Windows, macOS, and UNIX operating systems. *IntelliGenes* and its prerequisites can be readily deployed via GitHub or the Python Package Index (PyPI), with a preference for the utilization of our GitHub repository. It necessitates a Python version from 3.6 to 3.11 for proper functionality. *IntelliGenes* has been meticulously engineered to exhibit efficiency and adaptability, rendering it amenable for deployment on a spectrum ranging from personal computing devices to high-performance computing environments. Its functionality is contingent upon the utilization of multiple Python packages (“pandas,” “numpy,” “scikit-learn,” “xgboost,” “shap,” “matplotlib,” “scipy”). Further details of *IntelliGenes*’ s methodology are available in Supplementary Material S1, attached. The source code of *IntelliGenes* is publicly shared using GitHub and Code Ocean. To facilitate a better understanding of how to use. *IntelliGenes*, a user guide discusses resources on how to get started in Supplementary Material S2.

## 3 Results

The outcome of *IntelliGenes* includes the visual representations of SHAP values. Automated summary plots are generated representing the range of importance on the *x*-axis; near-zero SHAP values denote negligible impacts on predictive capabilities, negatively scored SHAP features help predict control patients, and positively scored features are efficient predictors of disease. In Supplementary Material S2, we have added examples results using a test dataset, which mainly reports ENSG00000139644 (TMBIM6) as a valuable predictor found for cases at low expression levels among patients with cardiovascular diseases (CVDs) and controls at high levels. In this biomarker’s I-Genes Profile, the feature is marked is underexpressed. In addition, we have reported a study (DeGroat *et al.* 2023a), where using *IntelliGenes*’ s methodology we have been able to discover novel biomarkers associated and predict CVDs with high accuracy. We have uncovered 18 transcriptomic biomarkers that are highly significant in the CVD population that were used to predict disease with up to 96% accuracy (DeGroat *et al.* 2023a).

## 4 Discussion

The development of an ML predictive engine that utilizes genetic biomarkers to assess the risk of complex disease in patients is still in its early stages. Despite the rapidly advancing interest in ML and the growing usage, there are no standard applications or tools available for nonexpert users especially without any computational backgrounds. Scientific researchers who wish to practice ML algorithms and create smarter biomedical solutions do not have applications to guide them in incorporating multi-genomic, clinical, and demographic data together for accurate research outcomes. Also, the use of single algorithm versus a combination of algorithms is another challenge to consider for more accurate results. We need ML applications for disease prediction, using diagnosis-pertinent biomarkers to discover a patient’s status using an ensemble of classifiers.

To predict disease with high accuracy, we first developed an ML application, i.e. Hygieia (DeGroat *et al.* 2023b). It is based on the RF for regression analysis and prediction without requiring hyperparameter tuning. We implemented the RF algorithm because in our comparative analysis and review study to investigate ML approaches using multi-genomic data (Vadapalli *et al.* 2022), we proved that it has been preferred over other models, especially when applied to relatively small datasets. We applied Hygieia to our AI/ML ready dataset, which was based on RNA-seq driven gene-expression, clinical and demographic data of patients with CVDs (Venkat *et al.* 2023). During our ML analysis using Hygieia, visible data clusters were observed for the genes highly correlated, downregulated and with altered expression in CVD patients compared to healthy individuals (Venkat *et al.* 2023). During our predictive analysis, we uncovered an interesting correlation between age, gender, race, and CVD diagnoses. We observed that age and gender appeared to have a high correlation in HF, while age and race were highly correlated in AF (Venkat *et al.* 2023). Our model was able to correctly classify individuals as CVD patients and predict CVDs with 95% accuracy. However, the scope of Hygieia was limited to the targeted disease specific genes, which we have overcome with the development of *IntelliGenes*, as it can analyze the complete transcriptome of patients based on the RNA-seq drive gene expression values. Molecular testing of these observations in independent experiments/patient populations and following up on their function with wet lab experimentation will lend credit, passing the extra line of validation.

*IntelliGenes* is a Findable, Accessible, Intelligent, and Reproducible (FAIR) ML pipeline with a unique combination of classical statistical methods and state-of-the-art ML algorithms to identify novel biomarkers and predict diseases. By integrating these approaches, we outperformed single algorithms, resulting in enhanced accuracy, deeper insights, and more precise predictions, essential for personalized early disease-risk detection in individuals. Through the convergence of statistical algorithms and machine learning classifiers, *IntelliGenes* offers high-accuracy, modular disease prediction and classification, enabling the discovery of novel disease-associated biomarkers and the development of gene–disease networks. It does not only hold the potential for personalized early detection of common and rare diseases in individuals, but also opens avenues for broader research using novel ML methodologies, ultimately leading to personalized interventions and novel treatment targets. Multiclass classification tasks require novel methodologies; we suggest that integrating patient demographics, transcriptomics, variants, and epigenomics can facilitate an unsupervised clustering approach that will allow us to map diseases onto patients through the extraction of these clusters’ most important features. In future, we look forward to improving our methodology by curating an unsupervised learning study that removes the labels to indicate status of health and allow the computer to cluster data points based on integrated gene expression and variant data along with clinical, demographics, and longitudinal data.

## Supplementary Material

btad755_Supplementary_Data

## Data Availability

The source code of *IntelliGenes* is available on GitHub (https://github.com/drzeeshanahmed/intelligenes) and Code Ocean (https://codeocean.com/capsule/8638596/tree/v1).

## Biographical note

W.D., A.B., and H.A. are Research Assistants at the Ahmed lab, Rutgers IFH/RWJMS. D.M. is the Lead Software Engineer at Rutgers IFH. S.Z. is the Bioinformatics Scientist at the Washington University School of Medicine in St Louis, and the former post-doctoral researcher at Rutgers Cancer Institute of New Jersey.

Z.A. is the Assistant Professor at the Department of Medicine/Division of Cardiovascular Diseases and Hypertension, Rutgers Robert Wood Johnson Medical School, and Rutgers Health. Z.A. is a Core Faculty Member at the Rutgers Institute for Health, Health Care Policy and Aging Research, at Rutgers, The State University of New Jersey. Furthermore, Z.A. is the Adjunct Assistant Professor at the Department of Genetics and Genome Sciences, School of Medicine, UConn Health, CT.
