# Supplementary material for: IntelliGenes: a novel machine learning pipeline for biomarker discovery and predictive analysis using multi-genomic profiles
Source: Bioinformatics. 2023 Dec 14;39(12):btad755. doi: 10.1093/bioinformatics/btad755 (PMC10739559; doi:10.1093/bioinformatics/btad755)
Supplement: btad755_Supplementary_Data [file btad755_supplementary_data.zip › Supplementary_Material_2_UserGuide.pdf]

## Supplementary Material# 2:

*IntelliGenes*: Installation, configuration, and user's guidelines

## Manuscript:

*IntelliGenes*: A novel machine learning pipeline for biomarker discovery and predictive analysis using multi-genomic profiles


## Authors

William DeGroat[1], Dinesh Mendhe[1], Atharva Bhusari[1], Habiba Abdelhalim[1], Saman Zeeshan[2], and Zeeshan Ahmed[1, 3, *]

## Affiliations

1. Rutgers Institute for Health, Health Care Policy and Aging Research, Rutgers University, 112 Paterson St, New Brunswick, 08901, NJ, USA.

2. Rutgers Cancer Institute of New Jersey, Rutgers University, 195 Little Albany St, New Brunswick, 08901, NJ, USA.

3. Department of Medicine, Robert Wood Johnson Medical School, Rutgers Biomedical and Health Sciences, 125 Paterson St, New Brunswick, NJ, 08901, USA.

***Corresponding author:** Zeeshan Ahmed, Rutgers Institute for Health, Health Care Policy and Aging Research, Rutgers University, 112 Paterson Street, New Brunswick, 08901, NJ, USA. (zahmed@ifh.rutgers.edu).

# Table of Contents


1. Installation ..... 3
  1.1 GitHub Installation ..... 3
  1.2 Python Package Index Installation ..... 3
2. Requirements ..... 3
  2.1 Software Requirements ..... 3
  2.2 Hardware Requirements ..... 3
3. User's Guide ..... 4
  3.1 Functions ..... 4
  3.1 CGIT Format ..... 4
  3.2 Results ..... 5
4. Data Availability ..... 6
5. Recorded tutorial ..... 6

# 1. Installation

*IntelliGenes* and its dependencies are easily installable using the Ahmed Lab's GitHub or the Python Package Index (PyPl). Using our GitHub is recommended. *IntelliGenes* depends on Python >=3.6.

## 1.1 GitHub Installation

*IntelliGenes* can be installed through our GitHub using the terminal. Follow the provided steps to install *IntelliGenes* and the package's dependencies:

```
# Clone IntelliGenes' GitHub Repository

git clone https://github.com/drzeeshanahmed/intelligenes.git

# Navigate to IntelliGenes

cd /intelligenes/

# Install IntelliGenes

pip install .
```

## 1.2 Python Package Index Installation

*IntelliGenes* can be installed through our PyPI using the terminal. Follow the provided steps to install *IntelliGenes* and the package's dependencies:

```
# Install IntelliGenes

pip install intelligenes
```

# 2. Requirements

*IntelliGenes* has been designed to be efficient and versatile. It is suitable for both local machines and high-performance computing environments. *IntelliGenes* depends on various Python packages.

## 2.1 Software Requirements

*IntelliGenes* depends on external Python packages. These packages will be installed alongside *IntelliGenes*: 'pandas', 'numpy', 'scikit-learn', 'xgboost', 'shap', 'matplotlib', 'scipy'.

## 2.2 Hardware Requirements

*IntelliGenes* requires computational muscle to make accurate predictions and discover novel biomarkers. Here, we recommend hardware requirements for running *IntelliGenes*:

- Processor: 2-core CPU
- Memory: 8GB RAM
- Storage: Sufficient disk space to store *IntelliGenes'* input and output files.

These specifications will change according to the users' dataset.

## 3. User's Guide

*IntelliGenes* offers a robust selection of tools to help users understand their multi-genomics datasets. *IntelliGenes* has been designed as an easy-to-understand pipeline for those at all levels of computational understanding.

### 3.1 Functions

*IntelliGenes* has three functions:

```
# Discover Biomarkers

igenes_select -i /data/cgit_file.csv -o /results/

# Disease Prediction & I-Genes Scores

igenes_predict -i /data/cgit_file.csv -f features_file.csv -o /results/

# IntelliGenes (Discovering Biomarkers & Predicting Disease)

igenes -I /data/cgit_file.csv -o /results/
```

These commands all users to write various flags that will tailor *IntelliGenes* to their exact needs:

```
# IntelliGenes Selection Help

Igenes_select --help

# IntelliGenes Prediction Help

Igenes_predict --help

# IntelliGenes Help

igenes --help
```

These various flags allow users to create their own ensemble model, generate *I-Genes Scores*, produce visualizations, normalize datasets, tune hyperparameters, and more. Breakdowns of the algorithms at work within *IntelliGenes* can be found in **Supplementary Material 1**.

### 3.1 CGIT Format

*IntelliGenes* requires a CGIT formatted dataset as an input. Examples of CGIT datasets can be found on our GitHub. The CGIT formatted dataset integrates demographics and transcriptomics:

- Columns contain demographic or transcriptomic biomarkers, while rows contain identifiers for individual patients.
- Demographics such as 'Age', 'Race', and 'Sex' should be integers (use EHR standards). These demographics are not required, as *IntelliGenes* works using only genomics/transcriptomics.
- There must be a 'Type' column, denoting a patient's status as an integer (use 0 or 1).

## 3.2 Results

*IntelliGenes* produces statistical and visual results, each output to the users' target directory. We have executed *IntelliGenes* on a sample dataset. The CGIT file and all results are available on GitHub:

cgit_file.csv_XX-XX-XXXX-XX-XX-XX-XX_Selected-Features.csv is a statistical output detailing the biomarkers found through the selector methods.

cgit_file.csv_ XX-XX-XXXX-XX-XX-XX-XX _RF-SHAP.png is a visual output that shows the SHapley Additive exPlanations (SHAP) plots for the Random Forest (RF) algorithm.

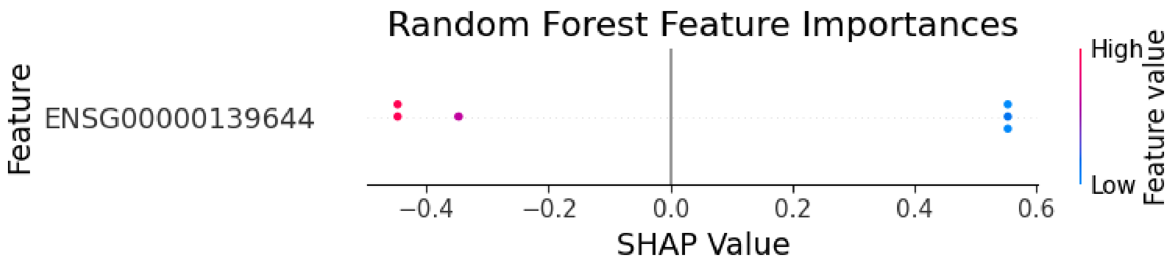


cgit_file.csv_ XX-XX-XXXX-XX-XX-XX-XX _SVM-SHAP.png is a visual output that shows the SHAP plots for the Support Vector Machine (SVM) algorithm.

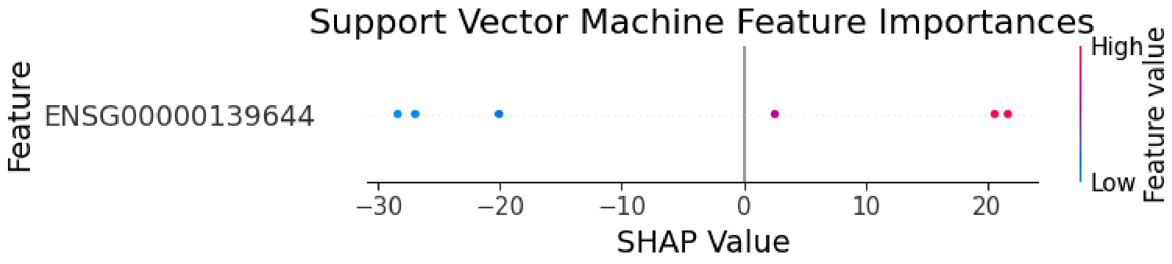


cgit_file.csv_ XX-XX-XXXX-XX-XX-XX-XX _kNN-SHAP.png is a visual output that shows the SHAP plots for the Random Forest (kNN) algorithm.

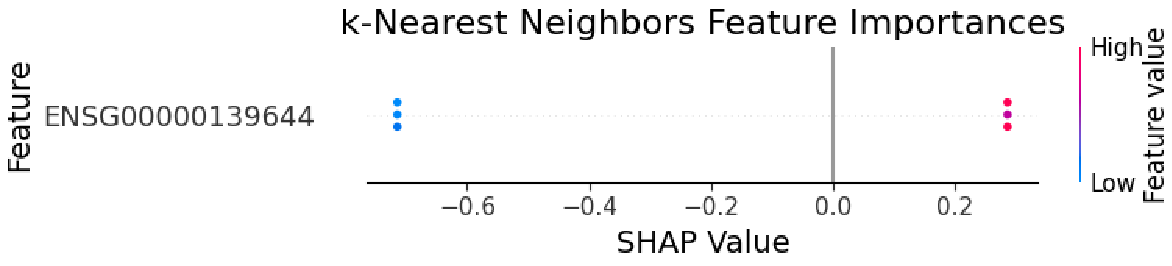


cgit_file.csv_ XX-XX-XXXX-XX-XX-XX-XX _XGB-SHAP.png is a visual output that shows the SHAP plots for the XGBoost algorithm.

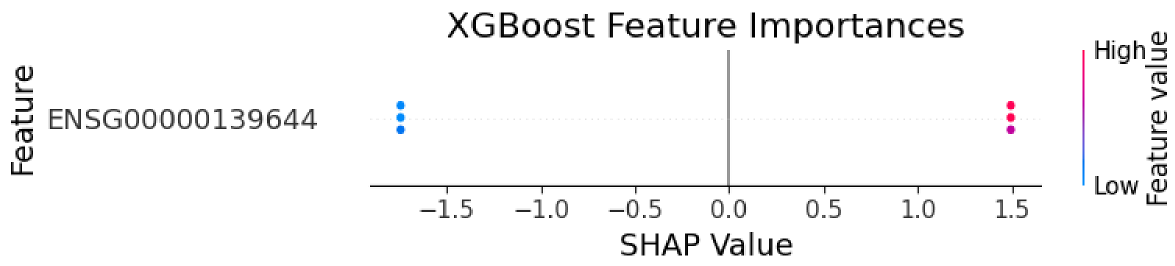


cgit_file.csv_ XX-XX-XXXX-XX-XX-XX-XX _MLP-SHAP.png is a visual output that shows the SHAP plots for the Multi-Layer Perceptron (MLP) algorithm.

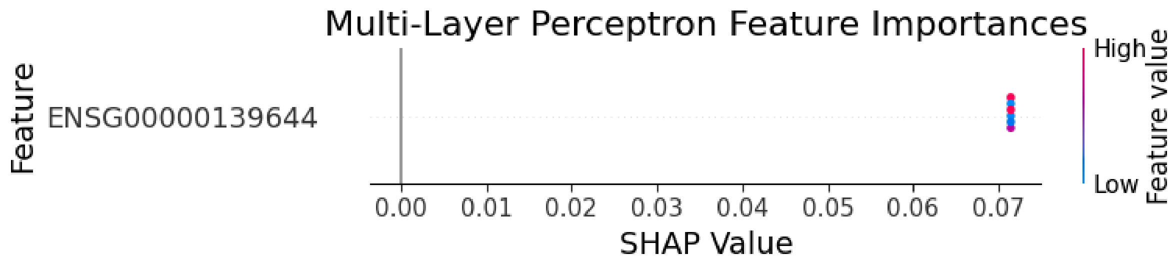


cgit_file.csv_XX-XX-XXXX-XX-XX-XX-XX_Classifier-Metrics.csv is a statistical output detailing the classifiers' performance in disease prediction. This includes accuracy, F1 scores, and ROC-AUC.

cgit_file.csv_XX-XX-XXXX-XX-XX-XX-XX_I-Genes-Score.csv is a statistical output detailing the *I-Genes* score of the biomarkers used in prediction. This score describes their relevance in the ensemble model.

## 4. Data Availability

The *IntelliGenes* source code and the data supporting this study are freely accessible on our GitHub page: *IntelliGenes* << https://github.com/drzeeshanahmed/intelligenes>>

## 5. Recorded tutorial

To better assist users in executing *IntelliGenes*, we have shared the recorded tutorial (online YouTube video) that includes following steps:

1. Command line configuration
**2.** Installation and verification of compatible Python
**3.** Installation and verification of git and pip
4. Download *IntelliGenes* from GitHub Repository
5. Navigation and installation of *IntelliGenes*
6. Executing *IntelliGenes* to discover biomarkers, disease Prediction, and I-Gene score.

URL to the online video tutorial:

< https://www.youtube.com/watch?v=LW6S1KujbTk&ab_channel=AhmedLab >
