# Supplementary material for: IntelliGenes: a novel machine learning pipeline for biomarker discovery and predictive analysis using multi-genomic profiles
Source: Bioinformatics. 2023 Dec 14;39(12):btad755. doi: 10.1093/bioinformatics/btad755 (PMC10739559; doi:10.1093/bioinformatics/btad755)
Supplement: btad755_Supplementary_Data [file btad755_supplementary_data.zip › Supplementary_Material_1_Method.pdf]

Supplementary Material# 1:

IntelliGenes: Methodology description

Manuscript:

IntelliGenes: A novel machine learning pipeline for biomarker discovery and predictive analysis using multi-genomic profiles

Authors

William DeGroat[1], Dinesh Mendhe[1], Atharva Bhusari[1], Habiba Abdelhalim[1], Saman Zeeshan[2], and Zeeshan Ahmed[1, 3, *]

Affiliations

1. Rutgers Institute for Health, Health Care Policy and Aging Research, Rutgers University, 112 Paterson St, New Brunswick, 08901, NJ, USA.
2. Rutgers Cancer Institute of New Jersey, Rutgers University, 195 Little Albany St, New Brunswick, 08901, NJ, USA.
3. Department of Medicine, Robert Wood Johnson Medical School, Rutgers Biomedical and Health Sciences, 125 Paterson St, New Brunswick, NJ, 08901, USA.

***Corresponding author:** Zeeshan Ahmed, Rutgers Institute for Health, Health Care Policy and Aging Research, Rutgers University, 112 Paterson Street, New Brunswick, 08901, NJ, USA. (zahmed@ifh.rutgers.edu).

1. Attribute Selection

1.1. Recursive Feature Elimination (RFE)

The RFE function is a wrapper-based feature selection algorithm that eliminates the least significant feature in every recursive iteration. RFE was used to eliminate biomarkers that do not have high significance to CVD and reduce the computational load for the analysis downstream [1]. The RFE function, part of the scikit-learn package, takes two parameters: a user-defined scoring metric and a predetermined number of features. In our study, we chose the scoring metric to be a Decision Tree (also from the scikit-learn package), and the number of features to be the top 10% from the original list of biomarkers, essentially performing a Quantile Analysis. The algorithm and the percentile of biomarkers can be tailored to suit different analyses. The correlation coefficient plays a crucial role in the RFE ranking system: the higher the coefficient, the higher the rank assigned to the gene, implying a stronger association between the gene and disease. It is important to note that a higher rank corresponds to a lower integer value. Our choice of the Decision Tree over the industry standard SVM was influenced by the inclusion of the Pearson Correlation, which already examines the linear relationships between biomarkers and disease [2]. The RFE results were depicted using a bar plot, where a smaller bar signifies a more significant gene. The selected biomarkers were compiled into a panda DataFrame. Despite its strengths, RFE has some limitations. It cannot quantify the correlation between biomarkers and lacks the ability to compute multivariate significance. Furthermore, due to its iterative nature, RFE has a high time complexity [3]. However, the time spent is justified by the algorithm's exceptional ability to eliminate weak, statistically insignificant features.

1.2. Pearson's Correlation

We apply Pearson correlation [5] to the gene list to determine each biomarker's linear correlation to disease. The Pearson correlation test is a statistical test that measures the strength of the linear correlation between two sets of data and follows the equation where *cov* refers to covariance and the equation to calculate covariance is also listed below.

$$p_{x,y} = \frac{cov(X,Y)}{\sigma_x \sigma_y} = \frac{E[XY] - E[X]E[Y]}{\sigma_x \sigma_y}$$

The Pearson correlation has been widely applied in classical genomics to calculate correlation coefficients between biomarkers and disease. Each biomarker is assigned a Pearson correlation coefficient and is collected into a panda DataFrame. We then apply a p-value significance test to the coefficient DataFrame to obtain the biomarkers with less than 0.05 p-value. One of the main limitations of the Pearson correlation test is the sensitivity to range differences between the biomarkers and their relation to disease. However, this can be accounted for by increasing the volume of data to reduce the range differences between biomarkers.

1.3. Chi-square Test

The Chi-square function (package: Scikit-learn) is implemented using a wrapper function called Select 'K' Best. This function examines the dependence between the test variable and the significant biomarkers. The chi-squared test has been applied widely in genomics for feature selection due to its application in multi-disease classification for genome-wide association studies (GWAS) and has been an industry standard since its inception [6]. The wrapper function requires two parameters: a scoring metric (in this case, chi-square) and 'k', which we set as ten. The chi-square test is performed using the equation:

$$x^2 = \Sigma \frac{(O_i - E_i)^2}{E_i}$$

Where x is the chi-squared value, the subscript refers to the current value in the feature at that given time, O is the observed value, and E is the mean value for that feature. Following the Chi-Square Test, a p-value significance test was performed on the results to identify the significant biomarkers. The main limitation of the Chi-square test is the number of Type I and II errors in small sample sizes. However, the rationale for implementing the chi-square test was to make our overall system predict better significant biomarkers in larger matrix sizes.

1.4. Analysis of Variance (ANOVA)

The Analysis of Variance, colloquially known as the ANOVA test, is a statistical formula used to compare variances across different groups. The ANOVA procedure uses a five-step approach to compute an f-statistic that determines the significance of a biomarker in tangent with a p-value significance test. We implemented the ANOVA test through the Select 'K' Best function (package: scikit-learn) and inputted 'f-statistic' as the scoring metric to assign the ANOVA algorithm to the function. Based on the scoring metric, the function then generates a score for each biomarker. The scoring metric is defined below, using a mathematical explanation in five steps:

Firstly, compute the correction terms for each biomarker:

$$C_x = \frac{(\sum X)^2}{N}$$

Use the values from (1) to compute the "Sum of Squares of Total":

$$SS_T = \sum X^2 - C_x$$

Use the values from (1) to compute the "Sum of Squares among groups":

$$SS_A = \frac{(\sum X)^2}{n} - C_x$$

Use the values from (2) and (3) to compute the "Sum of Squares within groups":

$$SS_w = SS_T - SS_A$$

Use the values from (3) and (4) to compute the F-ratio:

$$F = \left. \frac{SS_A}{k-1} \middle/ \frac{SS_W}{N-k} \right.$$

We then use the values from the f-statistic to calculate p-values and append those values to the DataFrame. The biggest limitation is the fact that if two groups of samples are of different sizes, then there is a direct issue with the strength and validity of the ANOVA test. However, with the inclusion of all the other algorithms that can handle imbalances in sample size, this limitation is not of concern to this study.

In our merged function, we select the biomarkers with a 0.05 or less p-value for the ANOVA, Chi-square, and Pearson Correlation test and show up in the top 10% of significant biomarkers in RFE. These biomarkers are supported by all the tests in the feature-selecting algorithm and thus filter out all the insignificant biomarkers.

2. Patient Classification

Biomarkers that were selected are utilized for patient prediction and classification tasks. We selected four algorithms: Random Forest (RF), Support Vector Machine (SVM), K-nearest neighbors (k-NN), and Extreme Gradient Boosting Decision Trees (XGB). All algorithms are applied to a hyperparameter tuning regimen, where the computer selects the best hyperparameters for each algorithm based on the input matrix. These four algorithms are ensembled using a Voting Classifier to curate a powerful predictive engine that can perform accurate classification that is specific to user-specified matrices.

2.1 Random Forest (RF)

RF [7] is a flexible and easy-to-use meta-classifier that combines the output of multiple decision trees to classify a patient. The subjects and the supported biomarker for each subject are inputted into the RF algorithm along with the health status for each subject. The algorithm computes a decision tree to classify patients based on their biomarker profile. Gini Index is often used to decide how the leaves of a tree should branch out.

$$Gini \ = 1 - \sum_{i=0}^{C} (p_i)^2$$

The algorithm is then hyperparameter-tuned to give a better prediction. The best decision tree from the forest is output and highlights the decision boundary (i.e., polynomial), which the algorithm uses to classify patients.

2.2 Support Vector Machine (SVM)

SVM [8] is a supervised machine learning algorithm that classifies data groups. A supervised learning algorithm is one that shows the input and desired output, and the algorithm analyzes the pattern between the input and output. SVM takes in the training dataset as an input and computes support vectors to classify patients based on their biomarker profile. The predicted class is computed using the following decision function:

$$w^T x + b$$

If the result is positive, the predicted class is a positive class (1); otherwise, it is the negative class (0):

$$\hat{y} = \begin{cases} 0, & w^T + b < 0 \\ 1, & w^T + b \geq 0 \end{cases}$$

The algorithm is then hyperparameter-tuned to garner a better prediction. The most important classification feature is output, highlighting the relative significance of each biomarker. The main limitation of SVMs is their inability to perform well when the data set is large. However, through a combination of algorithms, SVMs can be an integral part of an ML system when the input set is small.

2.3 Extreme Gradient Boosting (XGBoost)

XGBoost [9] is a scalable, distributed, gradient-boosted decision tree. Gradient boosting is utilized for classification tasks and it typically ensembles weak prediction models to create a stronger "meta" algorithm. The XGBoost algorithm takes the training dataset as an input and computes a decision tree to classify patients based on their biomarker profile. This algorithm is then hyperparameter-tuned to generate the best decision tree. The best decision tree for classification is then output, highlighting which biomarkers are used for classification by the algorithm. The main limitation of XGBoost is the performance on sparse and unstructured data. However, due to our robust data pre-processing function, we have been able to avoid this issue.

2.4 k-Nearest Neighbors (K-NN)

K-NN [10] is a machine learning algorithm that does not require any training phase. The K-NN algorithm determines the classification of a datapoint by majority voting amongst its 'k' nearest neighbors. As we need k nearest points, we first need to calculate the distance between the data points using the Euclidian distance formula, which is

$$d(x,y) = \sqrt{\sum_{i=0}^{n}(x_i - y_i)^2}$$

After given a positive integer k, K-NN assigns the observation closest to an observation and estimates the conditional probability that it belongs to that class using the following formula:

$$\Pr(Y = j|X = x_0) = \frac{1}{k}\sum_{i \in N_0} I(y_i = j)$$

The k-value was chosen based on iterating through all possible values of k and selecting the model with the highest accuracy. The hyperparameters were also tuned to generate the best model. The best model was selected, and the biomarkers used for classification were shown in a bar graph. The main limitation of KNN is the sensitivity to feature scaling. KNN calculates distances between instances to determine their similarity. If features have different scales, those with larger values can dominate the distance calculation, leading to biased results. It is essential to normalize or scale the features appropriately before applying KNN. However, KNN can adapt to changes in the training data without requiring complete retraining of the model, which is why it was selected for this analysis.

2.5 Multi-Layer Perceptron (MLP)

MLP [11] is a feed forward Artificial Neural Network consisting of fully connected neurons with a nonlinear kind activation function. It has input and output layers with one or more hidden layers stacked with neurons. Neurons in MLP can use any arbitrary activation function. As this is a feed forward algorithm, inputs are combined with initial weights in a weighted sum subjected to an activation function. Each layer transmits the output of its computation and internal representation of the data to the layer below it. This travels all the way from the output layer via the hidden layers. It would be impossible for the algorithm to figure out the weights that minimize the cost function if it merely computed the weighted sums in each neuron, transmitted findings to the output layer, and stopped there. There would be no actual learning if the algorithm performed only one computation.

2.6 Voting Classifier

All five algorithms were ensembled using the soft Voting Classifier, the class with the highest average probability of success is chosen as the final prediction. By combining each algorithm in this manner, the positives are accentuated while neutralizing the downsides for each algorithm. The function selects the appropriate algorithm for each matrix based on the attributes of that matrix, such as size, dimensions, and number of missing values.

2.7 I-Genes Score

I-Gene Score is metric developed using the Shapley Additive Explanations (SHAP) [12] and Herfindahl-Hirschman Index (HHI) [13]. SHAP is used to calculate Shapley values for each feature of the sample. Shapley value is the meaning of marginal contribution of each feature. Each of the Shapley values represents the impact of the feature on the prediction. Features with high SHAP value push towards one class and low SHAP value push towards the other class. Linear, Tree, Kernel SHAP are the types used for

each respective classifier. With help of these scores, a ranking is assigned to the genes. The higher the gene is ranked, the higher the probability of that gene helping detect disease.

Source Code availability

The source code of *IntelliGenes* is available on GitHub < https://github.com/drzeeshanahmed/intelligenes > and Code Ocean < https://codeocean.com/capsule/8638596/tree/v1 >

References

1. Kwak S. K., & Kim J. H. (2017). Statistical data preparation: management of missing values and outliers. Korean journal of anesthesiology, 70(4), 407–411. https://doi.org/10.4097/kjae.2017.70.4.407

2 Chen Q., Meng Z., Liu X., Jin Q. & Su R. (2018). Decision Variants for the Automatic Determination of Optimal Feature Subset in RF-RFE. Genes, 9(6), 301. https://doi.org/10.3390/genes9060301

3 Li Z, Xie W, Liu T (2018) Efficient feature selection and classification for microarray data. PLOS ONE 13(8): e0202167. https://doi.org/10.1371/journal.pone.0202167

5. Freedman, D., Pisani, R., & Purves, R. (2007). Statistics (international student edition). Pisani, R. Purves, 4th Edn. WW Norton & Company, New York.

6. Zhongxue C., Hanwen H., Hon K. T. N. (2012). Design and analysis of multiple diseases genome-wide association studies without controls. Gene, 510(1), 87-92. https://doi.org/10.1016/j.gene.2012.07.089

7. Ho, T. K. (1995). Random decision forests. In Proceedings of 3rd International Conference on Document Analysis and Recognition (pp. 278-282, Vol. 1). Montreal, QC, Canada. doi:10.1109/ICDAR.1995.598994.

8. Cortes, C., & Vapnik, V. (1995). Support-vector networks. Machine Learning, 20(3), 273–297.

9. Chen, T., & Guestrin, C. (2016). XGBoost: A Scalable Tree Boosting System. In Proceedings of the 22nd ACM SIGKDD International Conference on Knowledge Discovery and Data Mining. New York, NY, USA: ACM. 785–794. https://doi.org/10.1145/2939672.2939785

10. Mucherino, A., Papajorgji, P. J., & Pardalos, P. M. (2009). k-Nearest Neighbor Classification. In Data Mining in Agriculture. Springer Optimization and Its Applications, Vol. 34. Springer, New York, NY. https://doi.org/10.1007/978-0-387-88615-2_4

11. Haykin, S. (1999). Neural Networks: A Comprehensive Foundation. Prentice Hall.

12. Lundberg, S. M., & Lee, S. I. (2017). A Unified Approach to Interpreting Model Predictions. https://doi.org/10.48550/arXiv.1705.07874

13. Laine, C. R. (1995). The Herfindahl-Hirschman index: a concentration measure taking the consumer's point of view. The Antitrust Bulletin, 40(2), 423-432. https://doi.org/10.1177/0003603X9504000206
